# Elimination of lymphatic filariasis as a public health problem in Malawi

**DOI:** 10.1371/journal.pntd.0011957

**Published:** 2024-02-16

**Authors:** John Chiphwanya, Square Mkwanda, Storn Kabuluzi, Themba Mzilahowa, Bagrey Ngwira, Dorothy E. Matipula, Limbikani Chaponda, Paul Ndhlova, Prince Katchika, Chawananga Mahebere Chirambo, Philemon Moses, Justin Kumala, Martin Chiumia, Carrie Barrett, Hannah Betts, Joan Fahy, Maria Rebollo Polo, Lisa Reimer, Michelle C. Stanton, Brent Thomas, Sian Freer, David H. Molyneux, Moses J. Bockarie, Charles D. Mackenzie, Mark J. Taylor, Sarah Martindale, Louise A. Kelly-Hope

**Affiliations:** 1 Ministry of Health, Lilongwe, Malawi; 2 Malaria Alert Centre, Kamuzu University of Health Sciences, Blantyre, Malawi; 3 Centre for Health, Agriculture, Development Research, and Consulting, Blantyre, Malawi; 4 Centre for Neglected Tropical Diseases, Department of Tropical Disease Biology, Liverpool School of Tropical Medicine, Liverpool, United Kingdom; 5 World Health Organization, Geneva, Switzerland; 6 Department of Vector Biology, Liverpool School of Tropical Medicine, Liverpool, United Kingdom; 7 School of Community Health Sciences, Njala University, Bo, Sierra Leone; 8 Institute of Infection, Veterinary and Ecological Sciences, University of Liverpool, Liverpool, United Kingdom; Istituto Superiore di Sanità, ITALY

## Abstract

**Background:**

Lymphatic filariasis (LF) is a parasitic disease transmitted by mosquitoes, causing severe pain, disfiguring, and disabling clinical conditions such as lymphoedema and hydrocoele. LF is a global public health problem affecting 72 countries, primarily in Africa and Asia. Since 2000, the World Health Organization (WHO) has led the Global Programme to Eliminate Lymphatic Filariasis (GPELF) to support all endemic regions. This paper focuses on the achievements of the Malawi LF Elimination Programme between 2000 and 2020 to eliminate LF as a public health problem, making it the second sub-Saharan country to receive validation from the WHO.

**Methodology/principal findings:**

The Malawi LF Programme addressed the widespread prevalence of LF infection and disease across the country, using the recommended WHO GPELF strategies and operational research initiatives in collaboration with key national and international partners. First, to stop the spread of infection (i.e., interrupt transmission) and reduce the circulating filarial antigen prevalence from as high as 74.4% to below the critical threshold of 1–2% prevalence, mass drug administration (MDA) using a two-drug regime was implemented at high coverage rates (>65%) of the total population, with supplementary interventions from other programmes (e.g., malaria vector control). The decline in prevalence was monitored and confirmed over time using several impact assessment and post-treatment surveillance tools including the standard sentinel site, spot check, and transmission assessment surveys and alternative integrated, hotspot, and easy-access group surveys. Second, to alleviate suffering of the affected populations (i.e., control morbidity) the morbidity management and disability prevention (MMDP) package of care was implemented. Specifically, clinical case estimates were obtained via house-to-house patient searching activities; health personnel and patients were trained in self-care protocols for lymphoedema and/or referrals to hospitals for hydrocoele surgery; and the readiness and quality of treatment and services were assessed with new survey tools.

**Conclusions:**

Malawi’s elimination of LF will ensure that future generations are not infected and suffer from the disfiguring and disabling disease. However, it will be critical that the Malawi LF Elimination programme remains vigilant, focussing on post-elimination surveillance and MMDP implementation and integration into routine health systems to support long-term sustainability and ongoing success.

**Summary:**

Lymphatic filariasis, also known as elephantiasis, is a disabling, disfiguring, and painful disease caused by a parasite that infected mosquitoes transmit to millions of people worldwide. Since 2000, the Global Programme to Eliminate Lymphatic Filariasis (GPELF) has supported endemic countries such as Malawi in south-eastern Africa, to eliminate the disease as a public health problem. The Malawi National LF Elimination Programme has worked tirelessly over the past two decades to implement the GPELF recommended strategies to interrupt the transmission with a two-drug regime, and to alleviate suffering in patients with lymphoedema and/or hydrocoele through morbidity management and disability prevention. Additionally, the LF Programme has collaborated with national and international stakeholders to implement a range of supplementary operational research projects to address outstanding knowledge gaps and programmatic barriers. In 2020, the World Health Organisation validated that Malawi had successfully eliminated LF as a public health problem, making it the second country in sub-Saharan Africa to achieve this, which is remarkable given that Malawi previously had very high infection rates. The LF Programme now remains vigilant, putting its efforts towards post-elimination surveillance and the continued implementation of care for patients with chronic conditions. Malawi’s elimination of LF will ensure that future generations are not affected by this devastating disease.

## Introduction

Lymphatic filariasis (LF) is a parasitic neglected tropical disease (NTD) transmitted by mosquitoes, causing painful and disfiguring clinical conditions including lymphoedema and hydrocoele [1,2]. The disease imposes substantial physical, psychological, and economic burdens on endemic countries, communities, and the individuals it affects. The highest burden is concentrated in Asia and Africa, where *Wuchereria bancrofti* is the main parasite and *Culex* and/or *Anopheles* mosquitoes are the primary vectors. Over the past two and half decades, the World Health Organization (WHO) has led the Global Programme to Eliminate Lymphatic Filariasis (GPELF) to support the elimination of LF as a public health problem in all endemic countries, in accordance with the Resolution of the World Health Assembly WHA50.29 [3] and the recent NTD Road Map 2021–2030 targets [4].

The launch of GPELF drew international attention to this disabling disease, prompting endemic countries to commit to the cause and establish national LF elimination programmes. In Africa where the burden was considered to be high with an estimated 405.9 million people at risk in 2000 [5], the south-eastern country of Malawi took action and conducted several LF prevalence surveys to assess the extent of the problem across the country [6–8]. This initiative was fundamental, as it was known that LF was present in some communities in the northern region, but no surveys had been done since the late 1950s [9,10]. The nationwide prevalence surveys found that LF was endemic throughout most regions of the country, with particularly high prevalence in three remote rural districts; Karonga district in the northern region, and Chikwawa and Nsanje districts in the southern region.

In response to the widespread endemicity, the Malawi Ministry of Health established the National LF Elimination Programme in 2008 and followed the WHO GPELF strategy’s two main aims [1]:

1. to stop the spread of infection (interrupt transmission) through the annual distribution of preventive chemotherapy called mass drug administration (MDA) using a combination drug regimen, implemented for at least 5 years with at least 65% coverage of the total population, with supplementary vector control.
2. to alleviate the suffering of affected populations (control morbidity) through the implementation of morbidity management and disability prevention (MMDP) using the essential recommended package of care to manage lymphoedema and hydrocoele.

This paper aims to highlight the positive steps that the National LF Elimination Programme took between 2000 and 2020 to eliminate LF as a public problem in Malawi by implementing the WHO GPELF strategy and supplementary operational research activities with support from the Malawian government, as well as crucial technical and financial input from both national and international partners.

## Methods/Principal findings [results]

### Ethics statement

The Malawi LF Programme for LF Elimination activities is supported by the Ministry of Health and does not require ethical approval for programmatic activities related to the WHO recommended MDA and MMDP activities. The Liverpool School of Tropical Medicine (LSTM) obtained ethical clearance to support the WHO recommended MDA and MMDP activities (Research Protocols 12.22 and 11.89R). Additional ethics approval was obtained for the operational research activities described in this paper (i.e., published studies are excluded here as information is available in the related article). Formal written consent was obtained from all adult participants, and written consent from a parent or guardian for child participants.

○ Hotspot surveillance–National Health Sciences Research Committee (NHSRC Protocol 20/10/2611) and LSTM Research Protocol (19–116)
○ Easy Access Group (EAG surveillance) NHSRC Protocol (20/04/2563) and LSTM Research Protocol (20–029)
○ Lymphoedema management supply system (LyMSS)—NHSRC Protocol (15/3/1406) and LSTM Research Protocol (14.051).

### The GPELF strategy

The WHO GPELF strategy includes two main aims and a set of guidelines that are used by all endemic countries to eliminate LF as a public health problem [11–15]. Therefore, to demonstrate the positive steps that Malawi LF Elimination Programme took to eliminate the disease as a public health problem, here we present the methods and results together with the GPELF activities and several concurrent operational research activities, under the following sections:

- Malawi endemicity mapping
- Malawi LF Elimination Programme
- Implementation of interventions for the interruption of transmission
  ○ MDA; Supplementary interventions
- Epidemiological monitoring and post-treatment surveillance
  ○ Sentinel and spot check sites; Transmission Assessment Surveys (TAS); Supplementary assessmenst
- Morbidity management and disability prevention
  ○ Clinical case assessments and characteristics; Availability of treatment and services for lymphoedema and hydrocoele cases
- Resources and partnerships

### Malawi endemicity mapping

**Fig 1:**
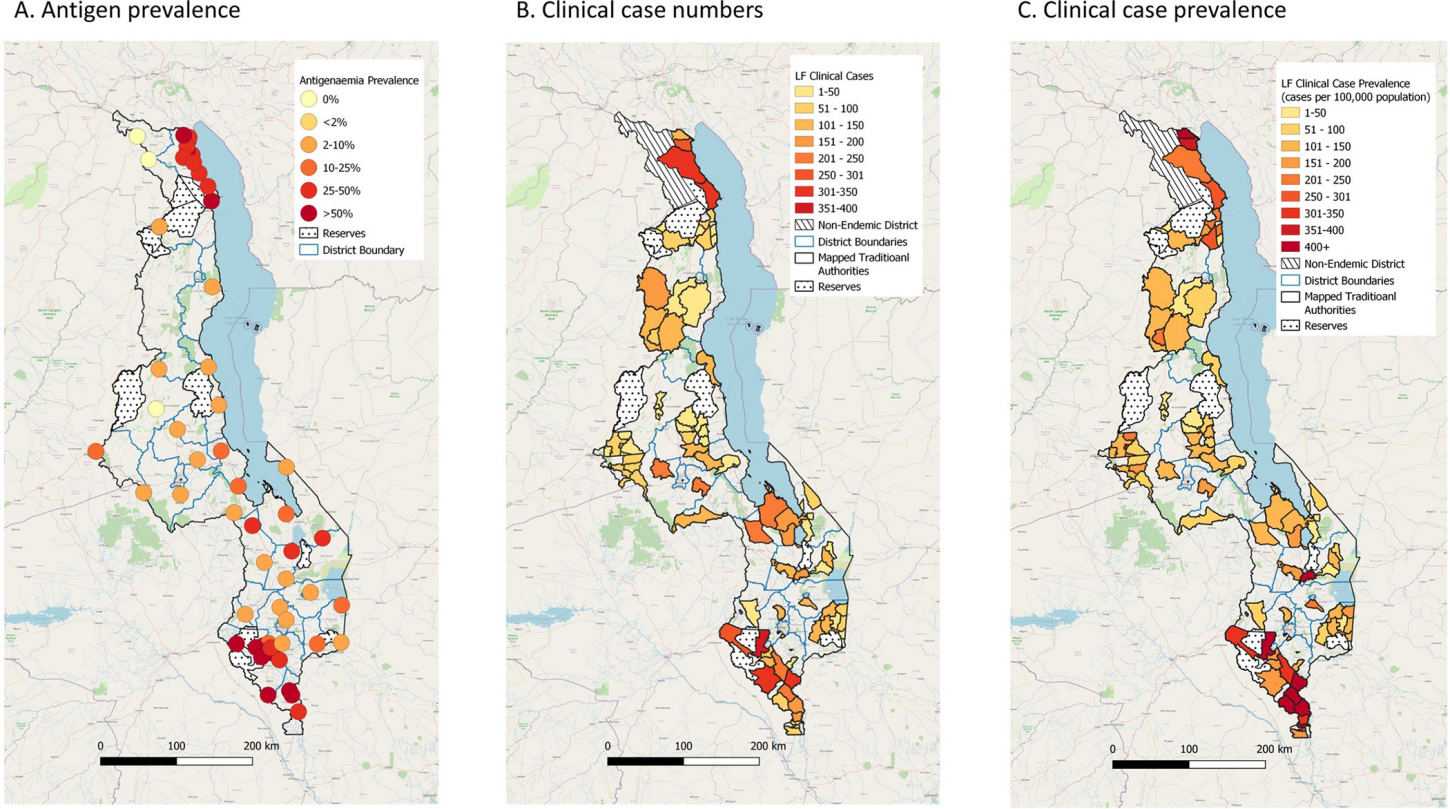
Distribution maps of LF prevalence of infection by sites (2002–2003) and clinical cases by Traditional Authority (2014–2020). A. Antigen prevalence. B. Clinical case numbers. C. Clinical case prevalence. Note: Maps were produced in QGIS mapping software (https://qgis.org) using the base layer from OpenStreetMap (https://www.openstreetmap.org/), and country administrative boundaries available from the Humanitarian Data Exchange [17].

In the early 2000s, three separate surveys were conducted in Malawi to determine the geographical extent of infection and disease [6–8]. The prevalence of *W*. *bancrofti* circulating filarial antigen was detected using the WHO recommended immunochromatographic test (ICT) diagnostic tool [16] and found that from the 54 communities surveyed the prevalence ranged from zero (Chitipa District) to 74.4% (Chikwawa District). Clinical case examinations in Karonga and Chikwawa Districts found higher than expected prevalence with up to 4.0% of people affected by lymphoedema and 18.0% of men by hydrocoele. Likoma Island was later surveyed in 2010 and found to be non-endemic. Maps in Fig 1A show the infection prevalence distribution and related data are available in S1 Table. All maps were produced in QGIS mapping software (https://qgis.org) using the base layer from OpenStreetMap (https://www.openstreetmap.org/), and country administrative boundaries available from the Humanitarian Data Exchange [17].

These surveys confirmed that 26 of 28 districts in Malawi were endemic as circulating filarial antigen prevalence rates were above the 1.0% threshold set by the WHO [12]. This led to the need for a National Elimination LF Programme to implement the GPELF strategy, which started in 2008 with the commitment and funding from the Malawi Ministry of Health and continued until 2020 with additional support from key national and international partnerships. A list of the partners and main roles is available in S2 Table.

### Malawi LF Elimination Programme

The Malawi LF Elimination Programme falls under the Preventative Health Services Directorate, Ministry of Health and is one of several other preventive chemotherapy focussed NTD programmes including onchocerciasis (also known as River Blindness), schistosomiasis (also known as Bilharzia), soil transmitted helminths (referred to as STH). Information on the organisational flow of the health system is presented in the text of the Malawi NTD Master Plan 2015–20 [18]. Each individual programme has a Programme Manager who reported directly to the Director of Preventative Health Services who in turn, reports to the Secretary for Health, Ministry of Health. The only NTD programme not in this Directorate is trachoma, which falls under the Directorate of Clinical Services.

The Malawi LF Elimination Programme officially began in 2008 and was led by the designated LF National Programme Manager, who was responsible for the planning and delivery of all aspects of the programme. The programme focussed on the 26 endemic districts identified in the early 2000s [6–8]. Each district represented one implementation unit (IU), which was based on WHO recommendations and used as the basis for making decisions about administrating activities related to the two main GPELF aims.

### Implementation of interventions for the interruption of transmission

#### Mass Drug Administration (MDA)

Malawi’s MDA programme included a combination of ivermectin (IVM: 200 μg/kg of body weight) and albendazole (ALB: 400 mg) to treat the entire at-risk population [11,12]. The drugs were donated by the Mectizan Donation Program (IVM) and GlaxoSmithKline (ALB) [19], and the logistics and transportation of these donated goods were managed by the LF Programme Manager and distributed from the Central Medical Stores in the capital city of Lilongwe, to the district-level headquarters, and onwards to all district health facilities.

One senior Health Surveillance Assistant (known as HSA) per health facility supervised a team who were responsible for estimating the number of drugs needed to reach the target population. This was achieved through community enumeration prior to MDA implementation activities and this information further helped to calculate MDA coverage rates and determine if the WHO threshold of 65% coverage of the total populations to interrupt transmission had been achieved [12]. Prior to the campaign, intensive social mobilisation activities took place to heighten community awareness and assist in reaching the required coverage. The senior Health Surveillance Assistant supervised the MDA campaign, which was implemented annually in December between 2008–2014 and typically lasted seven days with selected follow-up in areas of potentially low coverage. The IVM+ALB drug combination was distributed through a directly observed treatment (known as DOT) approach by volunteer Community Drug Distributors (known as CDDs), who were trained and supervised by their local Health Surveillance Assistants.

The first MDA activity was supported by the Ministry of Health in 2008 and implemented in nine southern districts that were co-endemic with onchocerciasis, which used the drug IVM as its primary preventive chemotherapy strategy. The LF MDA activities scaled up to reach all 26 endemic districts in 2009 and maintained until 2014. A summary of the national MDA implementation years, populations, and overall high reported coverage rates between 2008 and 2014 is shown in Table 1.

**Table 1:** Summary of national mass drug administration (MDA) implementation years, implementation units (IUs), populations, and reported coverage rates between 2008 and 2014.

Year	Type of MDA	No. of IUs covered	Total IU population	Reported no. of people treated	Reported MDA coverage
**2008**	IVM+ALB	8	3,358,816	2,704,323	80.5%
**2009**	IVM+ALB	26	13,053,083	10,805,518	82.8%
**2010**	IVM+ALB	27*	13,065,294	10,797,082	82.6%
**2011**	IVM+ALB	26	14,104,061	11,258,343	79.8%
**2012**	IVM+ALB	26	14,807,685	11,877,822	80.2%
**2013**	IVM+ALB	26	14,989,401	12,443,745	83.0%
**2014**	IVM+ALB	28*	15,309,468	12,751,028	83.3%

* Likoma Island (non-endemic district) was treated in 2010 with 78.4% reported coverage and again in 2014 with 89.9% reported coverage. Chitipa (non-endemic) was treated in 2014 with 84.6% reported coverage.

MDA coverage surveys to verify the reported rates were conducted between 2010 and 2014 in selected districts and all found to be above the required 65% threshold to interrupt transmission [12]. In Zomba, the WHO 30 cluster-survey protocol was conducted in 2010, and estimated coverage levels of 62.6% (95% Confidence intervals CI 56.5–68.7%) and 66.8% (95% CI 60.0–73.0%) in 2009 and 2010 respectively. An additional investigation into alternative coverage survey methods including convenience sampling in markets, and household surveys conducted by local headman and religious leaders found coverage at 74.3% (CI: 71.1%-77.4%), 76.3% (CI: 69.6%-83.0%), 77.8% (CI: 72.5%-83.1%), respectively [20]. In 2012, a WHO-cluster survey in Ntchisi district estimated coverage at 78.7%. In 2014, different survey methods were implemented in three districts; a WHO cluster-survey in Machinga district estimated coverage at 73% (95% CI 70.0–77.0%), a Lot Quality Assurance Sampling method in Balaka district estimated coverage at 86% (95% CI 79.0–93.0%), and a Probability Sampling with Segmentation method in Zomba district at 77% (95% CI 73.0–80%).

#### Supplementary interventions

The disease control and elimination programmes for malaria, onchocerciasis and soil transmitted helminths provided additional supplementary interventions that are likely to have helped to reduce LF transmission [18,21–23].

Malaria vector control interventions: The National Malaria Control Programme’s interventions include the distribution of insecticide treated/long-lasting insecticidal nets (ITN/LLINs) and indoor residual spraying (IRS) supported by national and international technical expertise and funding [22,23]. The periodic nationwide mass distribution of ITN/LLINs every three years from 2010, supplemented by routine distribution of nets at all antenatal clinics across the country. Further, there was localized indoor residual spraying (IRS) initially for two years (2009 to 2010) in a single district of Nkhotakota before scaling it up to a total of eight highly endemic districts close to Lake Malawi and the Lower Shire Valley with overall good coverage rates (>85%) until the programme was stopped in 2012 due to the emergence of pyrethroid insecticide resistance and lack of viable alternative insecticides. The main mosquito vectors of malaria and LF are similar and include the *Anopheles funestus* and *An*. *gambiae* complexes [24]. Therefore the distribution and impact of the vector control interventions for malaria, which have shown an overall reduction in malaria infection [25], are likely to have impacted the main LF vectors and helped to reduce the risk of transmission, particularly in areas where multiple interventions had been implemented [21].

Additional preventive chemotherapy interventions: The National Onchocerciasis Elimination Programme has distributed IVM MDA annually across eight endemic districts of the Southern Region of Malawi (Thyolo, Mwanza, Neno, Blantyre, Mulanje, Phalombe, Chiradzulu and Chikwawa) since 1997. From 2008 to 2014 these activities were integrated with the LF Programme, targeting all persons aged five years and above. The continued IVM MDA in these eight co-endemic districts at high levels, which has nearly eliminated onchocerciasis, is likely to have had an impact and reduced the risk of LF transmission [18,21].

The National Soil Transmitted Helminth Control Programme focuses on hookworm, roundworm, and whipworm parasites and has distributed ALB across selected population groups (e.g., school-aged children) in all 28 endemic districts since 2012. This has resulted in a reduction in the targeted parasitic infections, and has likely to have reduced the risk of LF transmission in these selected populations [18].

### Epidemiological monitoring and post-treatment surveillance

#### Sentinel and spot check sites

Sentinel site and spot check site surveys were conducted to assess the impact of MDA between 2008 (prior to starting MDA) and 2014 (prior to stopping MDA) using a combination of diagnostic methods to detect *W*. *bancrofti* microfilaria (MF) or circulating filarial antigen in the population. Table 2 summarises the data of each survey by each evaluation unit (EU), which comprised multiple adjacent districts with similar demographic and environmental characteristics as recommended by WHO guidelines [12]. In total 11 EUs were formed from the 26 endemic districts and used for pre-and post-MDA surveillance activities.

**Table 2:** Summary of impact assessments results by Evaluation Unit (EU) between 2008 and 2014.

EU Name	District Name	2008–2009 Baseline survey (MF)		2012 Mid-point survey (MF)		2014 Pre-TAS survey (CFA)		2014 Pre-TAS survey spot check (CFA)	
		Range of people tested/site (no. sites)	Site with highest % positives	Range of people tested/site (no. sites)	Site with highest % positives	Range of people tested/site (no. sites)	Site with highest % positives	Range of people tested/site (no. sites)	Site with highest % positives
**EU 1**	Chikwawa, Nsanje	342–450 (n = 2)	2.4%	312–470 (n = 2)	0.4%	300 (n = 2)	17.3%	300 (n = 6)	15.7%
**EU 2**	Blantyre,Mwanza, Neno, Chiradzulu	126–391 (n = 3)	1.6%	150–364 (n = 3)	2.7%	300 (n = 3)	0.7%	300 (n = 4)	3.0%
**EU 3**	Thyolo, Mulanje, Phalombe	210–263 (n = 3)	1.7%	220–400 (n = 2)	0.5%	300 (n = 3)	1.7%	300 (n = 2)	0.3%
**EU 4**	Zomba, Machinga	285–421 (n = 2)	0.7%	375–470 (n = 2)	0.2%	300 (n = 2)	4.3%	300 (n = 2)	2.7%
**EU 5**	Balaka, Mangochi	276–409 (n = 2)	1.5%	302–312 (n = 2)	0.0%	300 (n = 2)	0.0%	300 (n = 2)	0.0%
**EU 6**	Dedza, Ntcheu	206–245 (n = 2)	0.0%	-	-	300 (n = 2)	0.3%	-	-
**EU 7**	Lilongwe	**-**	-	-	-	-	-	300 (n = 1)	0.0%
**EU 8**	Mchinji, Kasungu	210–504 (n = 2)	2.9%	496 (n = 1)	0.0%	300 (n = 2)	2.3%	300 (n = 1)	0.3%
**EU 9**	Salima, Ntchisi Nkhotakota,Dowa	245–493 (n = 4)	1.8%	300–401 (n = 2)	0.0%	300 (n = 4)	0.0%	300 (n = 2)	0.3%
**EU 10**	Karonga, Rumphi	99–370 (n = 2)	9.1%	415 (n = 1)	0.5%	300 (n = 2)	1.3%	300 (n = 3)	2.3%
**EU 11**	Mzimba, Nkhata Bay	251–500 (n = 2)	0.0%	-	-	300 (n = 1)	0.0%	300 (n = 2)	1.0%

The baseline sentinel site surveys were conducted in 2008 (six sites) and 2009 (18 sites) prior to starting MDA. Participants over five years of age had finger prick blood samples collected from 22:00 to 01:00 to detect MF using the WHO microscopy standard protocol [12]. Overall MF prevalence rates ranged from 0% to 9.1% (Table 2). The first mid-point impact assessment was conducted in 15 of the 24 sentinel sites in 2012 and found that the overall MF prevalence had reduced to less than 3% in all sites.

In 2014, a pre-TAS assessment to determine if MDA could be stopped was conducted in 24 sentinel sites and spot check sites using the ICT diagnostic tool [16]. Overall, the prevalence was found to be less than 5%, in all but one EU which included Chikwawa and Nsanje districts (15.7%-17.3%). All people found to have an ICT positive test were subsequently treated using the standard IVM+ALB drug combination (Table 2). On the recommendation of the WHO, the LF Programme initiated the first of three TAS activities in 2014.

### Transmission Assessment Surveys (TAS)

The WHO TAS protocol was used for the post-treatment surveillance activities [12]. A school-based cluster method was used due to the high enrolment (>80%) of students in primary schools across Malawi. Three TAS activities were conducted between 2014 and 2018 across all 11 EUs. In each EU, children aged 6 and 7 years were selected at defined sampling intervals from randomly selected schools which was repeated for each TAS round. Children were tested for the presence of circulating filarial antigen using finger prick blood and ICT and/or the newer Filariasis Test Strip (FTS) diagnostic tool [16,26]. All antigen positive children were offered IVM +ALB treatment with their parent’s verbal consent. Family members were not followed up for testing or treatment.

**Fig 2:**
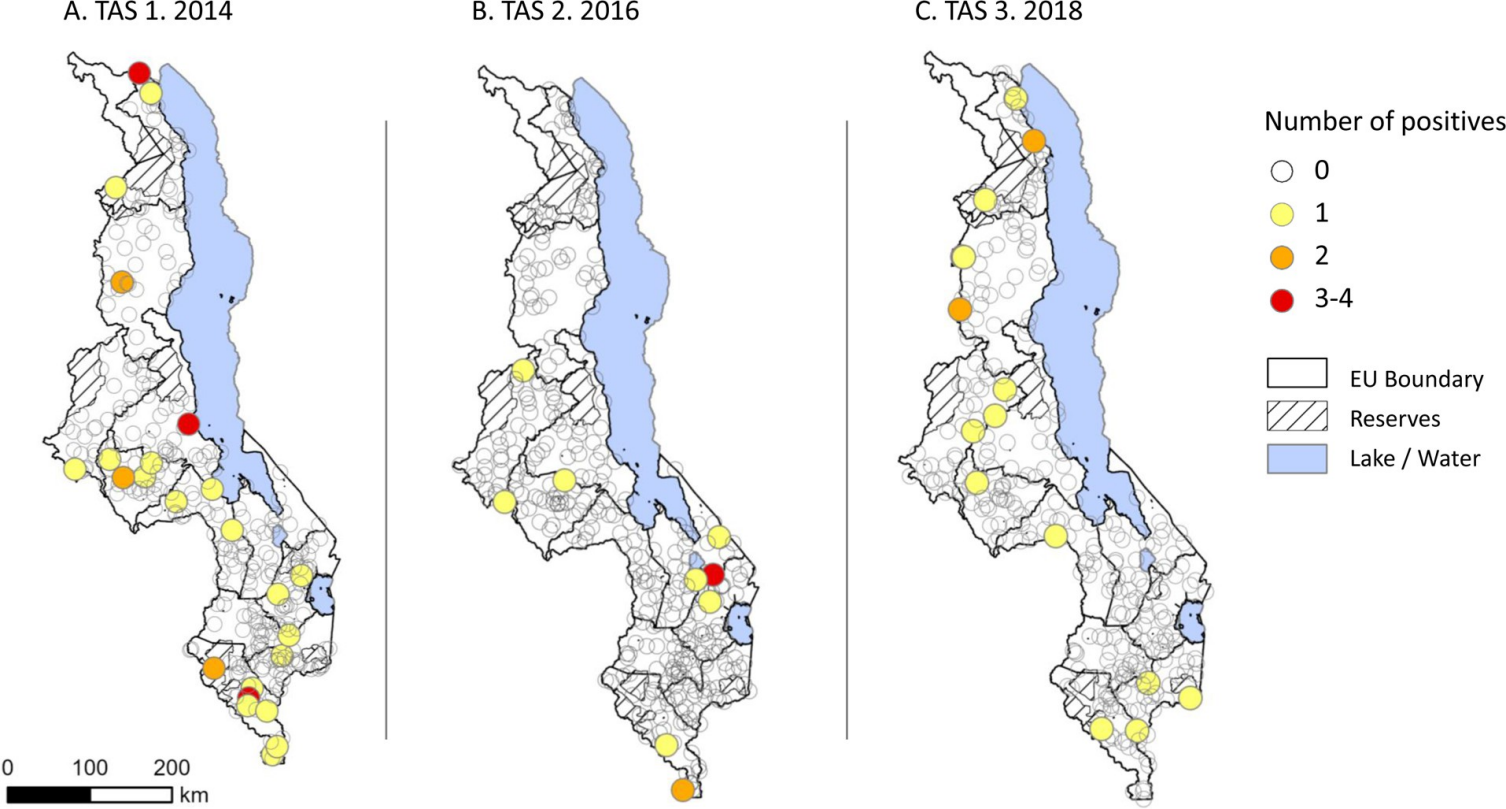
The distribution of Evaluation Units (EUs) and Transmission Assessment Survey (TAS) schools sampled by number of antigen positive children. A. TAS 2014. B. TAS 2. 2016. C. TAS 3. 2018. Note: Maps were produced in QGIS mapping software (https://qgis.org) and country administrative boundaries available from the Humanitarian Data Exchange [17].

TAS 3 included 20 additional schools as part of an enhanced protocol focussing on potential high-risk areas, which included the schools from TAS 1 and TAS 2 that had two or more positive children and schools on international borders where there is significant population movement with the neighbouring country. In addition, a supplementary surveillance assessment (described below) found infection in Chakanira village, Chikwawa district, so the village school was included in the TAS 3. Table 3 summarises the TAS results at EU level, and Fig 2 highlights the distribution of EUs and the schools sampled by the number of antigen positive children. All EUs passed the critical cut-off threshold (set at 18 or 20) indicating transmission has been interrupted.

**Table 3:** Summary of Transmission Assessment Survey (TAS) results between 2014 and 2018.

EU Name	District Name	TAS 1 Children surveyed	TAS 1 No. positives	TAS 2 Children surveyed	TAS 2 No. ICT positives	TAS 3 Children surveyed	TAS 3 No. positives	Total ICT/FTS positives
**EU 1**	Chikwawa, Nsanje	1,680	10 (3)*	1,693	3	1,717	1 (1)**	18
**EU 2**	Blantyre, Mwanza, Neno, Chiradzulu	1,677	1	1,926	0	1,697	1	2
**EU 3**	Thyolo, Mulanje, Phalombe	1,680	1 (0)*	1,700	0	1,765	0 (1)**	2
**EU 4**	Zomba, Machinga	1,674	2	1,724	1	1,698	0	3
**EU 5**	Balaka, Mangochi	1,671	0	1,694	6	1,701	0	6
**EU 6**	Dedza, Ntcheu	1,680	3	1,740	0	1,700	0 (1)*	4
**EU 7**	Lilongwe	1,673	6	1,727	0	1,717	1	7
**EU 8**	Mchinji, Kasungu	1,654	1	1,718	2	1,900	3	6
**EU 9**	Salima, Ntchisi Nkhotakota, Dowa	1,749	3	1,793	1	1,697	0	4
**EU 10**	Karonga, Rumphi	1,602	5	1,720	0	1,851	4	9
**EU 11**	Mzimba, Nkhata Bay	1,597	2	2,309	0	1,695	3 (2)**	7
**Total**		**18,337**	**34 (3)** *	**19,744**	**13**	**19,138**	**18 (5)** **	

* Filariasis Test Strip (FTS) diagnostic tool assessment in EU 1 and 3

** TAS 3 included 20 additional sites as part of an enhanced TAS protocol.

TAS 1 tested 18,337 children from 330 schools using the ICT diagnostic in 2014. An evaluation of the new FTS was conducted in two EUs as part of a wider international assessment for a new diagnostic for GPELF [26]. In total, 34 children from 25 schools in 10 EUs were found to be positive with the ICT diagnostic tool (three schools x3 children; three schools x2 children and 19 schools x1 child) and three children with FTS. The highest number of positives found was in EU 1 (n = 10 ICT; n = 3 FTS), which included the previously high endemic districts of Chikwawa and Nsanje. TAS 2 tested 19,744 children from 339 schools in 2016, with 13 children from nine schools found to be positive with the FTS diagnostic (one school x4 children; one school x2children and 7 schools x 1 child). The highest number of positives was found in EU 5. TAS 3 enhanced activity tested 19,138 children from 309 schools (289 standard; 20 additional) in 2018, found 18 children (13 standard; 5 additional) from 15 schools (11 standard; 4 additional) to be positive with FTS diagnostic (standard = two schools x2 children, eight schools x1 child; additional one school x2 children, 3 schools x1 child). Summary TAS result data by school sampled are available in S3 Table.

#### Supplementary assessments

The Malawi LF Elimination Programme worked collaboratively with a range of national and international partners to conduct additional assessments related to transmission and surveillance including i) vector control ii) entomological monitoring iii) integrated surveillance iv) targeted surveillance v) hotspot surveillance and vi) alternative surveillance. Summaries of the related assessments are described here, and summary results are available in Tables A-F in S4 Table.

Vector control: As part of the TAS 2 and TAS 3 programme activities described above, additional information on ITN/LLINs was collected. The children sampled were asked if their house had an ITN/LLIN (i.e., bed net asked in local language) and if yes, did they sleep under it the night before. The TAS 2 survey found 73.6% (range EU2 41.5%–EU7 72.1%) of children reported a bed net in their house and 61.7% (EU7 37.4%—EU4 91.5%) reported to have slept under it the night before the survey. The TAS 3 survey found 68.0% (range EU11 48.0%–EU1 90.9%) children reported a bed net in their house and 58.2% (EU31.6% - 83.0%) reported to have slept under it the night before the survey. These results indicate overall moderate to high ITN/LLIN coverage for this 6-7-year age-group of children, with a reduction in ownership and usage in TAS 3. The reason for this small change is not known as no additional questions were asked. It is possible it may be related to the timing of the national ITN/LLIN distributions. The information was shared with the National Malawi Control Programme so that low coverage areas may be followed-up according to protocols. A summary of the results is available in Table A in S4 Table.

Entomological monitoring. An entomological assessment was conducted between June and August 2017 in the high-risk districts of Chikwawa and Nsanje with support from national partners based at the Malaria Alert Centre of the University of Malawi. The study included five sites in Chikwawa District (Belo, Misili, Chakanira, Kumwembe and Mlooka) and two sites in Nsanje District (Gulumba and Mailosi), which were found to have high infection and/or clinical case rates in previous investigations. Mosquitoes were sampled from approximately 100 households across the seven sites using pyrethrum spray knockdown catches and Centers for Disease Control and Prevention (CDC) light traps [15]. Samples were counted, morphologically identified and members of the *Anopheles* species identified by polymerase chain reaction (PCR). Further, reverse transcription PCR (RT-PCR) was used to detect *W*. *bancrofti* parasites in a sub-sample of mosquitoes from each study site. Mosquito DNA was extracted and pooled into pools of five mosquitoes, a sample size that is in line with WHO guidelines [15]. The main LF vectors included *An*. *funestus* (n = 2832) and *An*. *gambiae* s.l (n = 215) and of these 38 *An*. *funestus* mosquitoes were found to be positive in the high-risk sites of Belo (n = 19/1848; 1.0%) and Chakanira (n = 14/953; 1.5%) in Chikwawa District. A summary of the laboratory methods and results are available in Table B in S4 Table.

Integrated surveillance: An arbovirus and LF integrated surveillance activity was conducted from November 2017 to January 2018 by the National NTD programme. Eight sentinel sites were established primarily to monitor arboviral infections due to a potential threat of dengue at the time. LF testing using FTS diagnostic tool was integrated into the activity. Testing was conducted at health facilities by trained staff and included the first 20 people who presented at the facility with acute febrile illness characterized by high fever, body pains and/or were malaria negative. A total of 1,196 people were tested (56.2% male) with three people found to be FTS antigen positive from Nkhotakota, Matawale in Zomba District and Ndirande in Blantyre city, and subsequently treated using the standard IVM+ALB drug combination. A summary of the results is shown in Table C in S4 Table.

Targeted surveillance: A household prevalence survey was conducted in six villages of Chikwawa district in 2013 based on reports of high numbers of lymphoedema or hydrocoele cases and varying sub-district levels of MDA coverage. In each village between 105 and 117 people were surveyed and infection measured using the ICT diagnostic tool and any positive people were followed up for MF testing. Additional information was collected on MDA intake in 2013, ITN/LLIN ownership as a supplementary intervention and if there were cases of clinical cases within the household.

A total of 795 individuals were surveyed and overall village MDA intake (range 69.2–90.2%), and ITN/LLIN (range 74.5–92.0%) coverage rates were high. In total, 15% of participants were ICT positive (12.4% females; 20.0% males). MF assessments were conducted in 66 ICT positive participants with 12% positive (9.0% females; 16.0% males). Median MF density was 22 MF/20 μL (range 0.3–58.5 MF/20 μL) in individuals who reported no MDA participation and 2.5 MF/20 μL (range: 0.7–8.0) in those who recalled participation in 2012. The percentage of clinical cases varied widely (lymphoedema 0.9–26.7%; hydrocoele 0 .0–3.4%). The village of Chakanira had the highest rates of infection (38.5% ICT; 19.4% MF) and clinical cases (26.7% lymphoedema; 3.4% hydrocoele) and was subsequently followed up during later TAS and MMDP activities. A summary of the results is available in Table D in S4 Table.

Hotspot surveillance: As part of a post-TAS surveillance activity a cross-sectional prevalence survey was conducted in the high-risk districts of Chikwawa and Nsanje in November 2020. Ten study sites considered to be potential transmission risk areas ‘hotspots’ were selected and grouped based on three different criteria; four sites with high baseline prevalence ‘Baseline hotspots’ (Belo, Mbnde, Gamba, Nchacha 18); four sites with high number of clinical cases ‘Morbidity hotspots’ (Chapananga, Lingawa, Kalemba, Mkango) and two sites with TAS positives ‘TAS hotspots’ (Muona, Nsenjere). In each site, between 300 and 350 people were targeted via household systematic random sampling and those who participated were tested using the FTS diagnostic tool. Additional information was collected on ITN/LLIN ownership (if no, state reason) and whether they slept under it the night before the survey, (if no, state reason) as a supplementary intervention. All FTS positive people were treated with the standard IVM+ALB drug combination.

A total of 3247 people (43.9% male; overall average age 25.2 years) were surveyed, with 20 people found to be FTS positive (0.6%) including 10 males (0.70% positive, average age 47.5 years) and 10 females (0.6% positivity, average age 35.6 years). Overall, there were similar prevalence ranges between the three hotspot groups; Baseline hotspots 0.6% (site range 0.0–1.7%); Morbidity hotspots 0.6% (site range 0.0–1.4%) and TAS hotspots 0.7% (site range 0.6–0.8%). The highest number of FTS positives were found in the Baseline hotspot of Belo, Chikwawa District (n = 5/301, 1.7%), and in the Morbidity Hotspot of Mkango, Nsanje District (5/365, 1.4%).

Overall, 70.7% people reported they owned an ITN/LLIN (n = 2297/3247; site range 53.0–83.8%) and 94.9% slept under it the night before (n = 2179/2297; site range 87.4–99.2%). Of the 950 people who did not own an ITN/LLIN, the main reason was they could not afford it (71.2%; n = 676/950) and of the 118 people who did not sleep under it, the main reason was that it was too hot (70.3% n = 83/118). The overall low *W*. *bancrofti* antigen prevalence indicated that transmission potential was low and likely to be further impacted by the overall high ITN/LLIN coverage. A summary of the results is available in Table E in S4 Table.

Easy Access Group surveillance: As part of a post-TAS surveillance, an operational research activity to determine the utility of screening an easy to access group (EAG) or population sub-group (i.e., pregnant women attending ante-natal clinic (ANC)) as a surveillance tool was conducted in Chikwawa District in September and October 2021 by national partners in collaboration with the LF Programme and international partners [27]. Two cross-sectional studies were conducted simultaneously. The first survey was conducted across 12 health facilities to screen pregnant women during their visit (i.e., ANC survey–survey 1). Each facility was grouped into transmission risk zones based on the distance from the Shire River, which is known to be a major breeding site associated with *Anopheles* vectors and shown to be correlated with disease [24,28]. The high-risk zone was 0-5km; moderate between 5-10km and low >10km from the riverbank. The second survey was conducted at the household level in the same catchment area of the sampled health facilities (i.e., Household survey–survey 2). In each site, approximately 70 households were selected at random from a central location and all household members aged above 5 years were invited to participate. For both surveys, participants were tested using the FTS diagnostic tool, and asked if the household had an ITN/LLIN. All positive people were followed up for MF testing and after were treated using the standard IVM+ALB drug combination.

For the ANC survey (survey 1), the high-risk zone included five clinics and 291 (26.8%) women; the medium-risk zone included four clinics and 470 (43.3%) women; and the low-risk zone included three clinics and 324 (29.9%) women. Of the 1,085 pregnant women tested (average age 24.1), only one from Chikwawa ANC was found to be FTS positive, but negative for MF (22 years of age, no ITN/LLIN). Overall, women reported moderate to low ownership of an ITN/LLIN (34.6%; site range 5.7%-58.8%).

For the Household survey (survey 2), a total of 2,900 individuals from 53 villages and 1,048 households were surveyed across the two districts. The high-risk zone included 1,107 (38.2%) individuals, the medium-risk zone 1,107 (34.0%) individuals; and the low-risk zone 808 (27.9%) individuals. Of the 2900 people tested, 4 were found to be FTS positive and two from high-risk zone (male aged 54; female aged 58) and two from the medium risk zone (males aged 25 and 45). Overall, people reported moderate to high ownership of an ITN/LLIN (53.0%; site range 27.6%-80.2%). A summary of the results from both surveys is available in Table F in S4 Table.

### Morbidity management and disability prevention (MMDP)

#### Clinical case estimates and characteristics

Historical studies published in 2002 found a high number of clinical manifestations in four villages in Karonga district (Northern Region) and two villages in the Lower Shire Valley (Southern Region) [7]. However, these studies only provided morbidity prevalence information for a small area. Therefore, as a first step to quantify the clinical burden on a larger scale, the LF Elimination Programme mobilised the Community Drug Distributors (known as CDDs) to collected additional information on the number of lymphoedema and hydrocoele cases during LF MDA implementation, and in 2011 a total of 5208 cases covering 11.7 million people were found: 3890 hydrocoele and 1318 lymphoedema cases, with the highest number reported from Chikwawa and Nsanje Districts. A summary of the results is available in Table A in S5 Table. This collection of data on morbidity cases was an important first step in understanding the burden. However, a study conducted in 2013 to determine whether this information collected during MDA accurately reflected the magnitude and distribution of lymphoedema cases, noting that in one catchment area of Chikwawa District, the actual number of cases of lymphoedema was over twice the number recorded in the MDA report [29]. This highlighted the need for improved methods of morbidity data collection in Malawi.

Active patient searching and mHealth data collection: In 2014, a mHealth real-time reporting tool (*MeasureSMS-Morbidity)* was piloted in three health centre catchment areas of Chikwawa District in which, trained Health Surveillance Assistants identified and reported all LF morbidity cases through active house-to-house patient searching. The methods are described elsewhere [29,30]. The mHealth tool allowed for the rapid collection and collation of information on LF patients including their location (community/health catchment name), age (number), gender (male, female), clinical condition (hydrocoele, lymphoedema, or both), stage of the condition (classified as mild, moderate, or severe as per WHO staging) and episodes of adenolymphangitis (ADL) also known as acute attacks (number in last 6 months) [13]. Following the successful pilot, and with funding from international partners between 2015–2021, the patient searching activities using the mHealth tool were scaled up across 23 districts. Seven districts were mapped completely (all health centre catchments) and 16 districts partially (selected health centre catchments) to obtain a geographical range of information from areas across the country with different levels of endemicity. The aim of the latter was to optimise limited resources given the expense of detailed mapping, and to provide an opportunity to develop models for unmapped areas (Traditional Authority level). This exercise facilitated the development of the first national LF clinical case database and LF morbidity map in Malawi, which was the first of its kinds in any endemic country in sub-Saharan Africa [31].

The clinical case mapping covered an area of over 33,000km^2^ populated by 5.6 million people, representing approximately one third of the country. Data were summarized by Traditional Authority (n = 90) and Fig 1B and 1C highlights the distribution of mapped areas, the number of clinical cases and prevalence per 100,000 population based on 2018 census data [32]. In total 8,856 clinical cases were identified, which comprised 6,333 hydrocoele cases in males (71.5%; average age 50.5); 2439 lymphoedema cases (27.5%; average age 50.9), and 84 hydrocoele and lymphoedema cases in men (0.95%; average age 58.8). The three known high endemic districts of Karonga (n = 1092), Chikwawa (n = 1386) and Nsanje (n = 1140) accounted for 40.9% (n = 3618/8856) of all cases reported. The number of cases by Traditional Authority ranged from 1 to 359, and the prevalence rates ranged from 3 to 1543 per 100,000 with the highest recorded in Chikwawa District. A summary of results is available in Table B in S5 Table.

**Fig 3:**
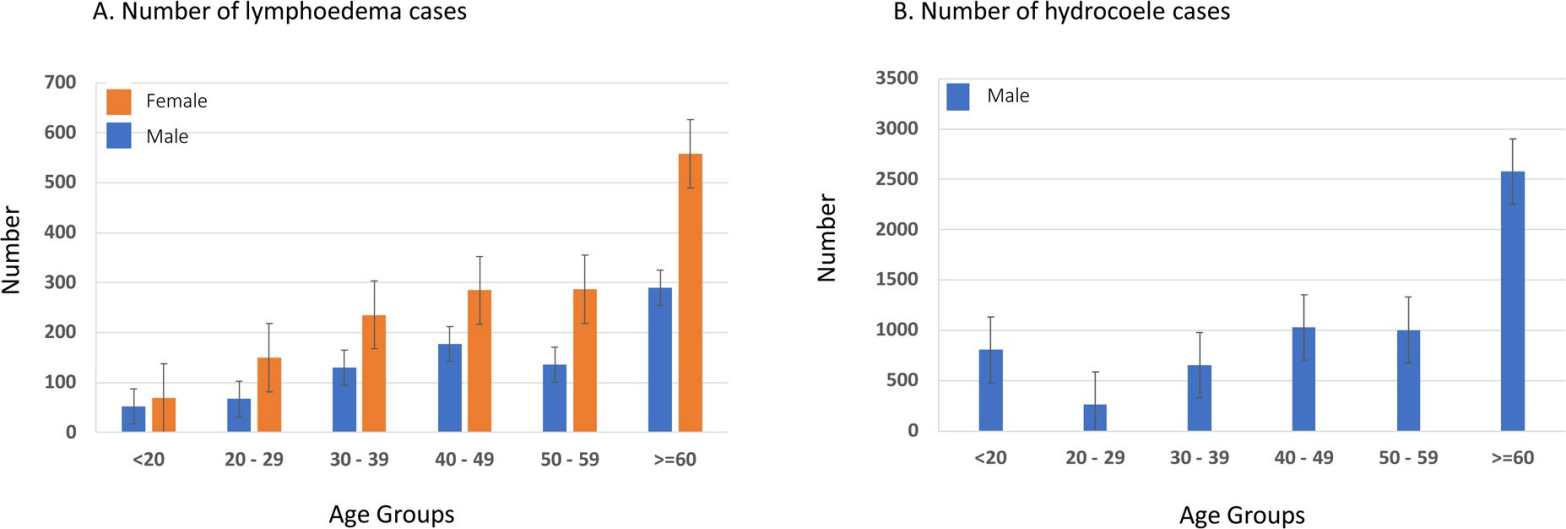
Summary of lymphoedema and hydrocoele cases by age group and gender. A. Number of lymphoedema cases. B. Number of hydrocoele cases.

The overall summary of the number of lymphoedema and hydrocoele cases by age group and gender is shown in Fig 3. For lymphoedema alone, more females (65.0%; n = 1585/2439) were reported than males (35.0%; n = 854/2439) and the number of cases increased by age for both genders with most cases aged >40 years (71.1%; n = 1734/2439) (Fig 3A). For hydrocoele, a similar increasing trend with age was found with most cases aged >40 years (72.8%; 4610/6333) Fig 3B). The hydrocoele cases reported in the <20-year age group were considered to be due to other causes but included here for reporting and specific follow-up purposes to confirm their diagnosis. A summary of the results is available in Table C in S5 Table.

**Fig 4:**
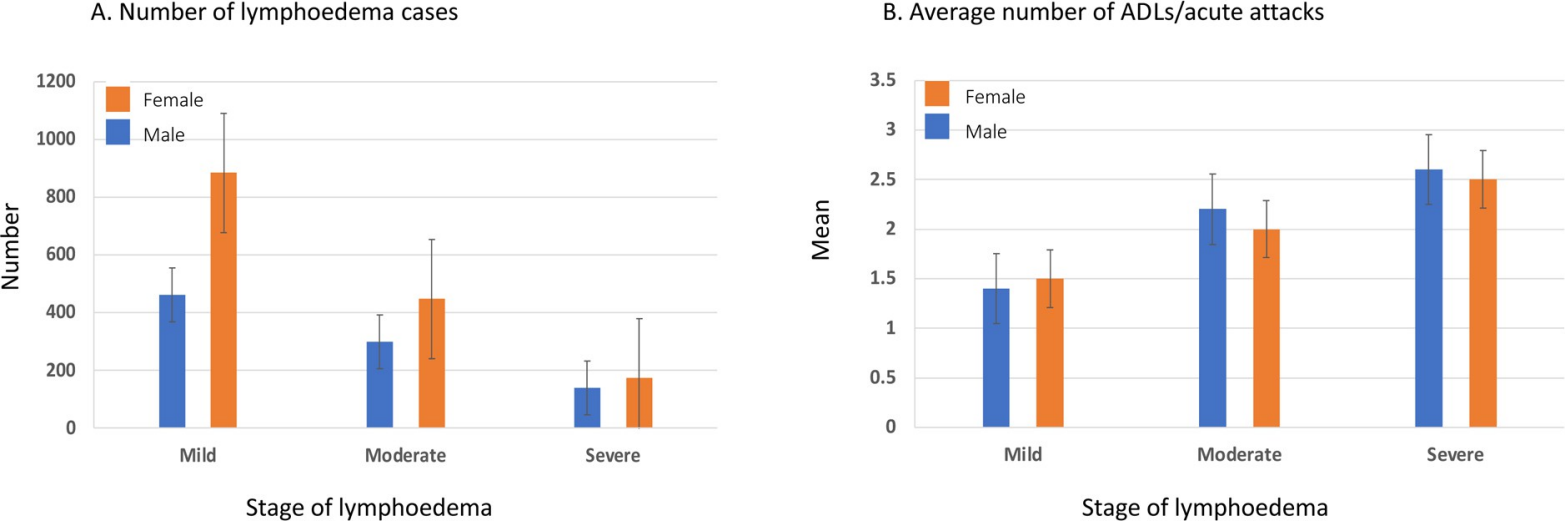
Summary of lymphoedema cases and ADLs/acute attacks by stage of condition and gender. A. Number of lymphoedema cases. B. Average number of ADLs/acute attacks.

The overall summary of lymphoedema cases and ADLs /acute attacks reported by stage of condition (mild, moderate, severe) and gender is shown in Fig 4. Overall, similar trends were found between males and females. For lymphoedema stage (n = 2403), there was a decreasing trend with most cases reported as mild (56.0%; n = 1345/2403), or moderate (31.0%, n = 746/2403) and fewer as severe (13.0%; n = 312/2403) (Fig 4B). For ADLs /acute attacks (n = 1957; note early data collections excluded this variable), the average (mean) number reported was 1.8 in the last 6 months. There was an increasing trend with severe lymphoedema cases (2.5) reporting a higher average number of acute attacks than cases with moderate (2.1) or mild (1.4) lymphoedema (Fig 4B). A summary of the results is available in Table D in S5 Table.

### Availability of treatment and services for lymphoedema and hydrocoele cases

Lymphoedema and adenolymphangitis (ADL) services: The provision of MMDP services for lymphoedema patients is based on WHO guidelines [13,14], and delivered through the primary care health system, including district health centres with their constituent clinics, dispensaries, and village clinics [18]. All health centres across the country provide free of charge services for patients who present with lymphoedema and ADL/acute attacks. A patient experiencing an ADL /acute attack may access additional services such as antibiotics or pain relief, at the health centre which is staffed by nurses and medical assistants or clinical officers (mid-level practitioners). Any patients with severe lymphoedema or experiencing complications that cannot be managed at the health centre, are referred to secondary and tertiary facilities for more specialized care.

General information on LF clinical conditions and care has been included in the routine government training of medical health workers. As part of the scale up of clinical case care between 2014 and 2021, all Health Surveillance Assistants and two staff from all 259 health centres involved in patient searching activities, and more than 4000 community health workers have been specifically trained on lymphoedema management and provided further training on lymphoedema home-based self-care to all patients identified in their local catchment.

In addition, several operational research projects were implemented in highly endemic districts to determine the impact of the clinical conditions and/or treatment strategies on patients affected by lymphoedema. Surveys on the quality of life highlighted how this disabling filarial condition negatively affected patient’s physical, psycho-social, mental health, and economic well-being [33,34], as well as caregivers [35]. In 2015, a tool for a lymphoedema management supply system (LyMSS) was developed [36], and piloted for six months, providing supplies for self-care to 62 patients. An interim follow-up brief survey of a sub-set of 13 patients was conducted. They self-reported an increase in frequency of limb care with availability of supplies, and that their clinical conditions had improved, showing a general reduction in swelling and/or pain. No independent clinical assessment was conducted at completion of the survey due to staff changes. In 2021, an enhanced self-care protocol [37] was implemented and showed substantial improvements in patient’s mental health via a survey tool [38]. The enhanced care protocol is now the main home-based approach being recommended for patients.

Health facility assessment: To determine the readiness to provide quality lymphoedema management services across the country an assessment was conducted in January and February 2019 using an adapted inspection protocol developed specifically for LF and supported by WHO [13,39]. A total of 56 facilities were surveyed. Half of the facilities had at least one facility staff member trained in lymphoedema management (50%; n = 28/56), and half had a system for identifying and quantifying the number of lymphoedema patients (50%; n = 28/56). Most facilities did not have a MMDP guideline available (3.6%; n = 2/56). Of the staff surveyed at each facility, 38 (67.9%) were able to correctly identify at least one sign or symptom of lymphoedema; 27 (48.2%) were able to correctly identify at least one sign or symptom of an ADL/acute attack; 27 (48.2%) were able to correctly identify at least one strategy to teach a lymphoedema patient to improve their condition and 28 (50.0%) knew how to treat a patient having an ADL/acute attack. In terms of the medicines and commodities available at the facility, a summary of the results is shown in Table 4.

**Table 4:** Summary of medicines and commodities available for lymphoedema at the 56 facilities surveyed in 2019.

Medicines and Commodities	Always available in sufficient quantities No. (%)	Most of the time available in sufficient quantities No. (%)	Never available No. (%)
Antiseptic (e.g., potassium permanganate or other anti-bacterial)	17 (30.4%)	5 (8.9%)	34 (60.7%)
Antifungal (e.g., potassium permanganate or Whitfield’s ointment)	20 (35.7%)	25 (44.6%)	11 (19.6%)
Analgesic or anti-inflammatory (e.g., paracetamol)	35 (62.5%)	19 (33.9%)	2 (3.6%)
Topical antibiotics (e.g., povidone-iodine, bacitracin)	34 (60.7%)	22 (39.3%)	0 (0%)
Oral antibiotics (e.g., amoxicillin, doxycycline)	39 (69.6%)	17 (30.4%)	0 (0%)
Injectable antibiotics (e.g., ampicillin, ceftriaxone)	42 (75%)	12 (21.4%)	2 (3.6%)
Ivermectin	6 (10.7%)	4 (7.1%)	46 (82.1%)
Albendazole	47 (83.9%)	9 (16.1%)	0 (0%)
Bucket or basin	38 (67.9%)	12 (21.4%)	6 (10.7%)
Soap	24 (42.9%)	23 (41.1%)	9 (16.1%)
Towels	3 (5.4%)	5 (8.9%)	48 (85.7%)
Gauze or cotton cloth	36 (64.3%)	19 (33.9%)	1 (1.8%)
Gloves	45 (80.4%)	11 (19.6%)	1 (1.8%)
Cold compress	4 (7.1%)	15 (26.8%)	37 (66.1%)

Hydrocoele services: The provision of MMDP services for hydrocoele patients is delivered at secondary level district hospitals by surgeons or clinical officers. There are 23 government district hospitals across the country, and in all these facilities patients can access hydrocelectomy services. In districts where there is no government hospital, gaps are met by faith-based mission hospitals. Complicated hydrocoele cases including persons with very large hydrocoeles and co-morbidities are referred to tertiary level hospitals. Malawi has four tertiary (central) hospitals which offer advanced and specialized care. Patients accessing hydrocoele surgery services are recorded in the theatre book.

The Malawi LF Elimination Programme has organised periodic hydrocoele surgery campaigns since 2008 with the aim to clear the backlog of cases in areas identified as having a high burden of clinical disease. These campaigns supplement the routine hydrocoele surgeries conducted throughout the country. Prior to hydrocoele campaigns, refresher training on the procedure is provided by regional surgeons to hospital staff and post-operative care training provided to community health workers including Health Surveillance Assistants. Since 2015 more than 1500 surgeries in high endemic districts have been performed, and research conducted to assess the impact of surgery on patients found an overall low rate of post-operative complications and significant improvements in patient’s quality of life [40], which also has a positive impact on their families, specifically caregivers [41]. Further an economic assessment found that the lifetime benefits of surgery to the individual, his family, and his community greatly outweigh the low cost of surgery (estimated at US$68 during campaigns) in Malawi [42]

Hydrocoele Surgery Facility Assessment Tool (HSFAT): To determine the readiness and quality of hydrocelectomy services, a new tool was developed and trialled in Malawi in February 2019 [43]. It used indicators from the WHO Service Availability and Readiness Assessment (SARA), and the WHO publication ‘Surgical approaches to the urogenital manifestations of lymphatic filariasis’ [14]. Readiness is a pre-requisite to ensure that facilities have the capacity to deliver services. Assessments included the presence of trained staff, guidelines, infrastructure, equipment, medicines, and laboratory tests. It was also deemed important to assess the hospitals current practice including the number of facilities available, availability of protocols, method of surgery, pre- and post-operative procedures, and methods of follow-up. The methods and results are described elsewhere [43]. A summary of key indicators from 24 hospitals surveyed across endemic districts in 2019 is shown in Table 5.

**Table 5:** Summary of key indicators from 24 hospitals surveyed in 2019.

Assessed Indicator	Present at facility No. (%)
**Total hospitals included in the analysis**	24
Water piped directly into hospital	24 (100%)
Electrical power supply (central, generator or solar)	24 (100%)
Electrical power supply backup	24 (100%)
Functional ambulance / other vehicle for emergency transportation	24 9100%
Currently performing surgeries	23 (95.8%)
All specified laboratory tests done onsite	23 (95.8%)
Appropriate functional method for sterilising/recycling instruments	24 (100%)
Appropriate functional method for disposing medical waste /sharps	24 (100%)
Clean, running water piped directly into the operating theatre	17 (70.8%)
Routinely use a surgical safety checklist	9 (37.5%)
Written protocols to distinguish complicated / uncomplicated cases	5 (20.1%)

### Resources and partnerships

The Ministry of Health financially supported the position of the LF Programme Manager within the Directorate of Preventative Health Service, all health personnel involved in MDA and MMDP implementation activities and the MDA implementation across nine endemic districts in 2008. The Malawi LF Elimination Programme collaborated with a range of national and international partners to achieve the successful elimination of LF as a public health problem. A list of the partners and main roles is available in S1 Table. Most of the financial support for MDA and MMDP implementation and operational research activities were from the Centre for Neglected Tropical Diseases (CNTD) at the Liverpool School of Tropical Medicine (LSTM) with a grant from the U.K. Foreign Commonwealth and Development Office (FCDO; formerly Department for International Development, DFID) and support from GlaxoSmithKline (GSK). The two drugs used for MDA implementation were donated by the Mectizan Donation Programme (IVM) and by GSK (ALB) [19].

## Discussion

This paper highlights the remarkable accomplishments of the Malawi LF Elimination Programme between 2000 to 2020 to eliminate LF as a public problem; the second country in sub-Saharan Africa, after Togo [44]. This achievement is noteworthy given that Malawi is one of the poorest countries in the world, and had widespread endemicity and high infection and disease rates across three remote rural districts in 2000. The programme achieved this milestone through the diligent implementation of the WHO-recommended MDA and MMDP initiatives and supplementary operational research projects. The success was made possible by a committed government and dependable programme managerial leadership and strategic partnerships with national and international partners who provided invaluable technical and financial support. All these factors have been identified as fundamental contributors to the success of national programmes [45]. Additionally, Malawi is a relatively peaceful and politically stable country compared with several other sub-Saharan countries that experience a variety of complex emergencies including extensive conflict, population displacement, infectious disease outbreaks (e.g. Ebola), and natural disasters, and urgently require additional strategies and support [46–48].

Notwithstanding the accomplishment, the Malawi programme encountered two broad challenges during elimination process, which limited the potential of various activities. The first challenge included the need for additional surveillance in low-prevalence areas as MDA was scaling-down. However, due the lack formal strategies available and limited funding, the programme piloted different approaches in selected high-risks areas i.e. enhanced TAS 3 and the integrated, targeted, hotspot and easy access group surveillance activities. Although the implementation process and results were useful, more could have been learned from a wider sample size and linked comparisons across different endemic zones. The timing of these additional activities was also affected by the COVID-19 pandemic [49], which further restricted opportunities to interact and share experiences with other countries and technical experts.

The second broad challenge was the need to understand and address the burden of clinical conditions across all endemic areas. While extensive case mapping has been conducted, and a modelled national map is now produced [31], a major challenge persists in addressing the large clinical case burden. This emphasises the need for significant funding for health working training, patient treatment and care, including the urgent scale-up of hydrocoele surgeries, affecting an estimated 21,000 men across the country [31]. Some areas, such as Chikwawa and Nsanje Districts, face additional challenges such as frequent flooding, which can inundate health centres and communities, displace populations, and re-direct limited resources. Currently no strategies exist specifically for NTD programmes to help prepare and respond to such emergencies and understand the impact.

The Malawi programme now maintains a vigilant stance, putting its efforts towards post-elimination activities that need integration with other national programme activities and/or incorporation into the routine health systems. Close connections with the National Soil Transmitted Helminth Control Programme and an understanding of the distribution and impact of ALB will help to identify areas potentially benefitting from the ongoing MDA–just as the LF MDA with IVM+ALB is expected to have provided a further impact on soil transmitted helminth prevalence and potentially scabies prevalence as shown elsewhere [50]. We also emphasise the need for sustained funding and committed partnerships from both domestic and international stakeholders to ensure continuing success of this public health endeavour, which will protect future generations. Further, we recommend that researchers continue to collaborate with the Malawi programme as there is much to learn about the post-elimination phase in the African context. Specific areas of focus may include:

i) Maximising the use of existing data to conduct additional epidemiological analysis and modelling to identify and examine potential areas of recrudescence or reintroduction.
ii) Enhancing collaboration with the malaria control programme to measure and monitor the impact vector control on LF vectors and potential residual transmission.
iii) Integrating surveillance into established ongoing monitoring and evaluation systems with attention on previously high-endemic areas and international border areas of risk. Introduce the use of new, more sensitive diagnostic tools as they become available.
iii) Counting and monitoring the number of clinical cases over time to demonstrate a reduction in new cases or ‘shrinking of the clinical case map’. Ensure all patients receive quality treatment and care, especially severe cases of lymphoedema and hydrocoele that are likely to require specialised or hospital care.
iv) Strengthening the health system processes, including health worker training, and data management.

## Conclusion

This paper provides a comprehensive overview of Malawi’s National LF Elimination Programme, highlighting the efforts undertaken to eliminate a neglected, disabling, and disfiguring disease as a public health problem from a widely endemic country. It serves as a guide for other national LF programmes, and the data included aims to inspire future research, in collaboration with the national programme, to address any challenges that may rise in the post-elimination phase.

## Supporting information

S1 STROBE Checklist Strobe statement. (DOC)

S1 Table Endemicity mapping results. (DOCX)

S2 Table National and international partners supporting the Malawi LF Elimination Programme and operational research activities. (DOCX)

S3 Table Transmission Assessment Survey (TAS) summary results. (XLSX)

S4 Table Summary of surveillance survey results. (DOCX)

S5 Table Summary of clinical case results. (DOCX)
