# Supplementary material for: Elimination of lymphatic filariasis as a public health problem in Malawi
Source: PLoS Negl Trop Dis. 2024 Feb 16;18(2):e0011957. doi: 10.1371/journal.pntd.0011957 (PMC10903958; doi:10.1371/journal.pntd.0011957)
Supplement: S1 Table — (DOCX) [file pntd.0011957.s002.docx]

**S1 Table. Endemicity mapping results**

| District (IU) | Endemic (Y/N) | Year | No. of survey sites | Range of people tested per site | Results from the site with highest % positives | Source |
| --- | --- | --- | --- | --- | --- | --- |
| Dedza | Y | 2003 | 1 | 64 | 7.8% | Source: Ngwira et al, 2007 [1] |
| Dowa | Y | 2003 | 1 | 72 | 5.6% | Source: Ngwira et al, 2007 [1] |
| Kasungu | Y | 2003 | 2 | 65 - 105 | 2.9% | Source: Ngwira et al, 2007 [1] |
| Lilongwe | Y | 2003 | 1 | 84 | 7.1% | Source: Ngwira et al, 2007 [1] |
| Mchinji | Y | 2003 | 2 | 98 - 99 | 18.2% | Source: Ngwira et al, 2007 [1] |
| Nkhotakota | Y | 2003 | 2 | 81 - 122 | 9.0% | Source: Ngwira et al, 2007 [1] |
| Ntcheu | Y | 2003 | 2 | 66 - 92 | 28.3% | Source: Ngwira et al, 2007 [1] |
| Ntchisi | Y | 2003 | 1 | 99 | 3.0% | Source: Ngwira et al, 2007 [1] |
| Salima | Y | 2003 | 2 | 73 - 78 | 21.9% | Source: Ngwira et al, 2007 [1] |
| Chitipa | N | 2003 | 2 | 77 - 85 | 0.0% | Source: Ngwira et al, 2007 This district was also re-mapped in 2011. 200 people in 2 sites were tested, with 1 positive result (0.5%) - data not presented here - Source: NTD Masterplan 2015-2020 [2] |
| Karonga | Y | 2000 | 12 | 42 - 102 | 57.8% | Source: Ngwira et al, 2007 [1] |
| Likoma | N | 2010 | 1 | 100 | 0.0% | Source: NTD Masterplan 2015-2020 [2] |
| Mzimba | Y | 2003 | 2 | 101 - 102 | 2.0% | Source: Ngwira et al, 2007 [1] |
| Nkhata Bay | Y | 2003 | 2 | 103 - 104 | 7.8% | Source: Ngwira et al, 2007 [1] |
| Rumphi | Y | 2003 | 2 | 72 - 82 | 9.8% | Source: Ngwira et al, 2007 [1] |
| Balaka | Y | 2003 | 1 | 53 | 35.8% | Source: Ngwira et al, 2007 [1] |
| Blantyre | Y | 2003 | 1 | 77 | 6.5% | Source: Ngwira et al, 2007 [1] |
| Chikwawa | Y | 2000 | 9 | 59 - 196 | 79.1% | Source: Ngwira et al, 2007 [1] |
| Chiradzulu | Y | 2003 | 1 | 81 | 7.4% | Source: Ngwira et al, 2007 [1] |
| Machinga | Y | 2003 | 1 | 70 | 4.3% | Source: Ngwira et al, 2007 [1] |
| Mangochi | Y | 2003 | 3 | 82 - 92 | 25.6% | Source: Ngwira et al, 2007 [1] |
| Mulanje | Y | 2003 | 2 | 69 - 78 | 18.8% | Source: Ngwira et al, 2007 [1] |
| Mwanza | Y | 2003 | 1 | 64 | 4.7% | Source: Ngwira et al, 2007 [1] |
| Neno | Y | 2003 | 1 | 64 | 4.7% | New district formed from Mwanza after the 2003 mapping survey. Therefore, Mwanza data included here - Source: Ngwira et al, 2007 [1] |
| Nsanje | Y | 2000 | 3 | 84 - 148 | 66.7% | Source: Ngwira et al, 2007 |
| Phalombe | Y | 2003 | 1 | 78 | 24.4% | Source: Ngwira et al, 2007 |
| Thyolo | Y | 2003 | 1 | 95 | 6.3% | Source: Ngwira et al, 2007 |
| Zomba | Y | 2003 | 1 | 57 | 3.5% | Source: Ngwira et al, 2007 |

**Reference**

1. Ngwira BM, Tambala P, Perez AM, Bowie C, Molyneux DH. The geographical distribution of lymphatic filariasis infection in Malawi. Filaria J. 2007;6: 12. doi:10.1186/1475-2883-6-12
2. Malawi NTD Master Plan 2015-2020. 2014 pp. 1–102. Available: https://espen.afro.who.int/countries/malawi
