# Supplementary material for: Elimination of lymphatic filariasis as a public health problem in Malawi
Source: PLoS Negl Trop Dis. 2024 Feb 16;18(2):e0011957. doi: 10.1371/journal.pntd.0011957 (PMC10903958; doi:10.1371/journal.pntd.0011957)
Supplement: S2 Table — (DOCX) [file pntd.0011957.s003.docx]

**S2 Table. National and international partners supporting the Malawi LF Elimination Programme and operational research activities.**

| Partner Name* | Activities Supported | Geographical Area Supported |
| --- | --- | --- |
| WHO | Endemicity Mapping - seroprevalence | Countrywide |
| MOH & CNTD, LSTM | MDA - Operational Costs | All LF endemic districts |
| MDP | MDA Drug Donation - Ivermectin | All LF endemic districts |
| GSK | MDA Drug Donation - Albendazole | All LF endemic districts |
| CNTD, LSTM & CDC | MDA Coverage Surveys | Ntchisi, Machinga, Balaka & Zomba |
| CNTD, LSTM | M&E – Sentinel Sites, TAS | All LF endemic districts |
| WHO | Diagnostic Test Donation (Filariasis Test Strip) | All LF endemic districts |
| CNTD, LSTM, & GSK | Morbidity Mapping | All LF endemic districts |
| MOH & CNTD, LSTM | Morbidity Training of Clinical Staff | Countrywide |
| MOH & CNTD, LSTM | Hydrocoele Surgeries | Countrywide |
| CNTD, LSTM | Operational Research Projects | Chikwawa & Nsanje districts |
| WHO Country Office | Technical Support | Countrywide |
| College of Medicine, Malawi | Technical Support | Countrywide |
| National Statistics Office, Malawi | Technical Support | Countrywide |
| COR NTD | EAG surveillance |  |
| GSK | Mapping /enhanced care studies |  |
|  |  |  |

* Names in full:

WHO- World Health Organization; MOH- Ministry of Health, Malawi; CNTD, LSTM– Centre for Neglected tropical Diseases, Liverpool School of Tropical Medicine, UK; MDP – Mectizan Donation Programme; GSK. GlaxoSmithKline; CDC – Centres for Disease Control and Prevention, Atlanta, USA; COR NTD – Coalition for Operational Research on Neglected Tropical Diseases, Atlanta, USA
