# Supplementary material for: Elimination of lymphatic filariasis as a public health problem in Malawi
Source: PLoS Negl Trop Dis. 2024 Feb 16;18(2):e0011957. doi: 10.1371/journal.pntd.0011957 (PMC10903958; doi:10.1371/journal.pntd.0011957)
Supplement: S4 Table — (DOCX) [file pntd.0011957.s005.docx]

**S4 Table. Summary of surveillance survey results.**

**Table A. Summary of data on ITN/LLIN ownership and use collected during the during Transmission Assessment Survey (TAS) 2 and TAS 3.**

| **EU Name** | **District**  **Name** | **TAS 2 (2016)** | | **TAS 3 (2018)** | |
| --- | --- | --- | --- | --- | --- |
|  |  | **% owning bed net**  (No.surveyed) | **% sleeping under bed net** | **% owning bed net**  (No. surveyed) | **% sleeping under bed net** |
| **EU 1** | Chikwawa,  Nsanje | 67.9%  (n=1693) | 59.6% | 90.9%  (n=1717) | 83.0% |
| **EU 2** | Blantyre, Neno, Mwanza,Chiradzulu | 62.3%  (n=1926) | 60.5% | 72.0%  (n=1697) | 63.4% |
| **EU 3** | Thyolo, Mulanje, Phalombe | 81.7%  (n=1700) | 72.1% | 70.0%  (n=1765) | 67.4% |
| **EU 4** | Zomba,  Machinga | 92.0%  (n=1724) | 91.5% | 72.1%  (n=1698) | 64.5% |
| **EU 5** | Balaka,  Mangochi | 78.4%  (n=1694) | 59.5% | 77.9%  (n=1701) | 62.6% |
| **EU 6** | Dedza,  Ntcheu | 78.5%  (n=1740) | 58.5% | 63.9%  (n=1700) | 53.9% |
| **EU 7** | Lilongwe | 41.5%  (n=1727) | 37.6% | 61.5%  (n=1717) | 49.0% |
| **EU 8** | Mchinji,  Kasungu | 74.3%  (n=1718) | 61.2% | 59.0%  (n=1900) | 50.5% |
| **EU 9** | Salima, Ntchisi Nkhotakota, Dowa | 73.5%  (n=1793) | 62.0% | 57.2%  (n=1697) | 47.5% |
| **EU 10** | Karonga,  Rumphi | 73.7%  (n=1720) | 52.0% | 71.4%  (n=1851) | 62.0% |
| **EU 11** | Mzimba,  Nkhata Bay | 84.1%  (n=2309) | 63.5% | 48.0%  (n=1695) | 31.6% |
| **Average** |  | 73.6%  (n=19,744) | 61.7% | 68.0%  (n=19,138) | 58.2% |

**Table B. Summary of mosquito genera tested, and mosquitoes detected carrying infective L3 *Wuchereria bancrofti* across the seven study sites**

| District | Site | *An. funestus* | | *An. gambiae* | | *Culex sp* | | *Mansonia* | |
| --- | --- | --- | --- | --- | --- | --- | --- | --- | --- |
|  |  | **No**  **tested** | **No.**  **positive** | **No**  **tested** | **No.**  **positive** | **No**  **tested** | **No.**  **positive** | **No**  **tested** | **No.**  **positive** |
| Chikwawa | Belo | 1848 | 19 | 58 | 0 | 349 | 3 | 71 | 2 |
| Chikwawa | Chakanira | 953 | 14 | 3 | 0 | 14 | 0 | 1 | 0 |
| Chikwawa | Misili | 7 | 0 | 44 | 0 | 204 | 0 | 67 | 0 |
| Chikwawa | Mlooka | 1 | 0 | 7 | 0 | 652 | 0 | 1 | 0 |
| Chikwawa | Kumwembe | 17 | 0 | 103 | 0 | 1,103 | 0 | 104 | 0 |
| Nsanje | Gulumba | 2 | 0 | 0 | 0 | 233 | 0 | 46 | 0 |
| Nsanje | Mailosi | 3 | 0 | 0 | 0 | 15 | 0 | 31 | 0 |
| Overall total | | **2,831** | **33** | **215** | **0** | **2,570** | **3** | **321** | **2** |

Laboratory methods were in line with WHO guidelines [15] available at [**https://www.who.int/publications/i/item/9789241505642**](https://www.who.int/publications/i/item/9789241505642)

*Morphological processing*

Mosquito samples were counted and identified to genus level using morphological identification keys and their abdominal status was recorded whilst still fresh. Sibling species within *An. gambiae* species complex and *An. funestus* group of mosquitoes were identified using polymerase chain reaction (PCR).

*Anopheles species identification*

QIAamp DNA minikit (Qiagen, Hilden, Germany) was used to extract DNA samples for species identification and parasite detection. Primers (Universal, GA, QD and AR) for *Anopheles gambiae* complex and primers (UV, FUN, VAN, RIV, PAR, RIVLIKE, LEES) for *Anopheles funestus* complex were used together with *An. gambiae* and *An. funestus* controls. For mosquito identification, a conventional PCR was used and the reactions were as follows: single cycle of 95°c for 5 min then 30 cyclic conditions of 95°C for 30sec, then 50°C for 30 seconds followed by 72°C for 40 sec. The samples were then held at 4°C until visualization on gel electrophoresis where sample bands were compared to positive control bands plus DNA ladder.

*Detection of Wuchereria bancrofti*

To detect *W. bancrofti* infective larval stages (L3), mosquito DNA was pooled into pools of 5 mosquitoes. From each single DNA tube, 2µL of DNA were transferred into a single and mixed thoroughly. In qPCR, 12.5 µL of Taqman Universal PCR MasterMix was used together with probe (FAM-TAMRA) and the forward and reverse primers. The total reaction volume was 25µL and thermal cycling conditions for the PCR were 95⁰C for 10 minutes (holding stage) followed by 40 cycles of 95⁰C for 15 seconds, 60⁰C for 60 seconds.

**Table C. Summary of number of people tested and found *Wuchereria bancrofti* antigen positive as part of an integrated arbovirus and LF surveillance activity across eight sentinel sites.**

| District | Health Facility | Surveillance Start Date | Surveillance End Date | No. Positive Results | No. Negative Results | No. Invalid Results | Total Tests |
| --- | --- | --- | --- | --- | --- | --- | --- |
| Karonga | Karonga DHO | Nov 2017 | Jan 2018 | 0 | 106 | 14 | 120 |
| Mzuzu | Mzuzu Central Hospital | Nov 2017 | Jan 2018 | 0 | 152 | 8 | 160 |
| Nkhotakota | Nkhotakota DHO | Nov 2017 | Jan 2018 | 1 | 96 | 2 | 99 |
| Lilongwe | Lilongwe DHO | Nov 2017 | Jan 2018 | 0 | 259 | 11 | 270 |
| Machinga | Machinga DHO | Nov 2017 | Jan 2018 | 0 | 115 | 0 | 115 |
| Zomba | Matawale Health Centre | Nov 2017 | Jan 2018 | 1 | 134 | 21 | 156 |
| Blantyre | Ndirande Health Centre | Nov 2017 | Jan 2018 | 1 | 147 | 0 | 148 |
| Chikwawa | Chikwawa DHO | Nov 2017 | Jan 2018 | 0 | 128 | 0 | 128 |
| Overall total | | | | **3** | **1137** | **56** | **1196** |

**Table D. Summary of targeted sites, number of people sampled, MDA participation, *Wuchereria bancrofti* antigen (ICT) and MF positive and vector control ownership in 2013**

Table 4D.1 MDA participation and LF prevalence

| Village | Total surveyed | % Female | MDA ever | MDA in 2012 | Mean MDA reported | | ICT+ (%) | MF+ (of ICT+) |
| --- | --- | --- | --- | --- | --- | --- | --- | --- |
| Bereu | 148 | 55 | 79.1 | 73.6 | | 2.0 | 17.6 | 6.7 |
| Chakanira | 133 | 73 | 76.7 | 69.2 | | 2.0 | 38.5 | 19.4 |
| Chipwaila | 127 | 67 | 90.6 | 79.5 | | 2.4 | 4.1 | nd |
| Lauji 2 | 124 | 72 | 92.7 | 87.9 | | 2.3 | 4.1 | 0 |
| Makwira | 132 | 64 | 94.7 | 90.2 | | 1.9 | 5.4 | nd |
| Ndakwira | 131 | 61 | 81.5 | 74.0 | | 2.3 | 10.8 | 6.7 |

nd= not done

Table 4D. 2 Household survey results for LF morbidity and LLIN coverage

| Village | Total surveyed | % households positive for lymphoedema† | % households positive for hydrocoele† | % with LLIN |
| --- | --- | --- | --- | --- |
| Bereu | 110 | 7.3 | 1.8 | 74.5 |
| Chakanira | 116 | 26.7 | 3.4 | 92.2 |
| Chipwaila | 105 | 1.0 | 0 | 80.9 |
| Lauji 2 | 111 | 0.9 | 1.8 | 81.1 |
| Makwira | 110 | 3.6 | 1.8 | 91.8 |
| Ndakwira | 117 | 6.0 | 0 | 80.3 |

†Adults only, no children were reported to have lymphedema or hydroc0ele

**Table E. Summary of hotspot sites, number of people sampled, *Wuchereria bancrofti* antigen positive and vector control ownership and use**

| Site selection | Sample | Age | *Wuchereria bancrofti* antigen positive | | | | Vector control | |
| --- | --- | --- | --- | --- | --- | --- | --- | --- |
| Hotspot type and site | **No. tested** | **Average** | **No. positive male** | **No. positive female** | **Total No.**  **positive** | **Total**  **positive**  **percent** | **ITN/LLIN**  **own**  **percent** | **ITN/LLIN**  **use**  **percent** |
| Baseline hotspots |  |  |  |  |  |  |  |  |
| Belo, Chikwawa | 301 | 25.8 | 2 | 3 | 5 | 1.66 | 80.4 | 99.2 |
| Mbnde, Chikwawa | 315 | 24.2 | 0 | 0 | 0 | 0.00 | 83.8 | 95.1 |
| Gamba, Nsjane | 311 | 24.1 | 1 | 0 | 1 | 0.32 | 70.4 | 97.7 |
| Nchacha 18, Nsanje | 310 | 28.2 | 1 | 0 | 1 | 0.32 | 77.1 | 92.5 |
| Baseline sub-total | **1237** | **25.5** | **4** | **3** | **7** | **0.56** | **77.9** | **96.1** |
|  |  |  |  |  |  |  |  |  |
| Morbidity hotspots |  |  |  |  |  |  |  |  |
| Chapananga, Chikwawa | 315 | 20.8 | 0 | 0 | 0 | 0.00 | 67.6 | 98.1 |
| Lingawa, Chikwawa | 329 | 24.6 | 1 | 1 | 2 | 0.61 | 54.7 | 96.7 |
| Kalemba, Nsanje | 315 | 27.3 | 1 | 0 | 1 | 0.32 | 53.0 | 89.9 |
| Mkango, Nsanje | 365 | 27.3 | 2 | 3 | 5 | 1.37 | 71.8 | 87.4 |
| Morbidity sub-total | **1324** | **25.1** | **4** | **4** | **8** | **0.60** | **62.1** | **92.7** |
|  |  |  |  |  |  |  |  |  |
| TAS hotspots |  |  |  |  |  |  |  |  |
| Muona | 366 | 24.9 | 1 | 2 | 3 | 0.82 | 72.6 | 98.9 |
| Nsenjere | 320 | 24.9 | 0 | 2 | 2 | 0.63 | 76.6 | 93.1 |
| TAS sub-total | **686** | **24.9** | **1** | **4** | **5** | **0.73** | **74.5** | **96.1** |
| Overall total | **3247** |  | **10** | **10** | **20** | **0.62** | **70.7** | **94.9** |

Table F. Summary of risk zones and sites, number of people sampled, *Wuchereria bancrofti* antigen positive and vector control ownership by ANC survey and Household survey

| Site selection | ANC Survey | | | | Household survey | | | | |
| --- | --- | --- | --- | --- | --- | --- | --- | --- | --- |
| Risk zone and site | **No. tested** | **Average**  **Age** | **FTS No. Positive** | **ITN/LLIN**  **Percent** | **No tested** | **Mean**  **Age** | **Female** | **ITN/LLIN Percent** | **FTS No. Positive** |
| High risk |  |  |  |  |  |  |  |  |  |
| Chikwawa | 63 | 24.7 | 1 | 42.9 | 198 | 27.9 | 57.1 | 66.2 | 0 |
| Maperera | 71 | 23.9 | 0 | 49.3 | 329 | 27.2 | 42.5 | 62.6 | 1 |
| Mfera | 43 | 23.0 | 0 | 44.2 | 187 | 29.5 | 50.3 | 80.2 | 1 |
| Nsanje | 80 | 24.7 | 0 | 58.8 | 185 | 26.0 | 56.8 | 27.6 | 0 |
| Tengani | 105 | 24.4 | 0 | 16.2 | 208 | 27.9 | 56.7 | 31.3 | 0 |
| Sub-total | **291** | **24.4** | **1** | **40.1** | **1107** | **27.7** | **54.4** | **45.5** | **2** |
|  |  |  |  |  |  |  |  |  |  |
| Medium risk |  |  |  |  |  |  |  |  |  |
| Makhuwira | 172 | 23.7 | 0 | 37.8 | 525 | 26.4 | 49.1 | 51.8 | 2 |
| Masenjere | 79 | 24.7 | 0 | 19.0 | 44 | 29.3 | 50.0 | 50.0 | 0 |
| Mbenje | 95 | 23.7 | 0 | 10.5 | 215 | 27.2 | 53.0 | 32.1 | 1 |
| Ndamera | 53 | 26.2 | 0 | 5.7 | 201 | 27.2 | 79.5 | 28.4 | 0 |
| Sub-total | **470** | **24.2** | **0** | **42.3** | **985** | **26.9** | **51.5** | **57.4** | **3** |
|  |  |  |  |  |  |  |  |  |  |
| Low risk |  |  |  |  |  |  |  |  |  |
| Beleu | 84 | 24.5 | 0 | 38.1 | 187 | 27.3 | 54.0 | 43.9 | 0 |
| Hunger | 159 | 23.5 | 0 | 45.3 | 421 | 27.6 | 54.6 | 42.5 | 0 |
| Kalemba | 81 | 24.0 | 0 | 40.7 | 200 | 27.1 | 55.5 | 36.5 | 0 |
| Sub-total | **324** | **23.9** | **0** | **23.3** | **808** | **27.4** | **54.7** | **58.7** | **0** |
| Overall total | **1085** | **24.1** | **1** | **34.6** | **2900** | **27.3** | **53.5** | **53.0** | **5** |

Note: Data are available from COR NTD Data Portal: <https://doi.org/10.15139/S3/LDDYU9>.

Each facility was grouped into transmission risk zones based on the distance from the Shire River, which is known to be a major breeding site associated with *Anopheles* vectors and shown to be correlated with disease. The high-risk zone was 0-5km; moderate between 5-10km and low >10km from the riverbank.
