# Supplementary material for: Elimination of lymphatic filariasis as a public health problem in Malawi
Source: PLoS Negl Trop Dis. 2024 Feb 16;18(2):e0011957. doi: 10.1371/journal.pntd.0011957 (PMC10903958; doi:10.1371/journal.pntd.0011957)
Supplement: S5 Table — (DOCX) [file pntd.0011957.s006.docx]

**S5 Table. Summary of clinical case results**

**Table A. Summary of clinical cases identified during by Community Drug Distributors (CDDs) during Mass Drug Administration activities across Malawi in 2011**

| EU Name | District  Name | Population | Lymphoedema cases | | Hydrocoele cases | | Total |
| --- | --- | --- | --- | --- | --- | --- | --- |
|  |  |  | **Number** | **Per 100,000 population** | **Number** | **Per 100,000**  **population** |  |
| EU 1 | Chikwawa, Nsanje | 637,944 | 396 | 62.1 | 1007 | 157.9 | 1403 |
| EU 2 | Blantyre, Mwanza, Neno, Chiradzulu | 1,293,051 | 109 | 8.4 | 160 | 12.4 | 269 |
| EU 3 | Thyolo, Mulanje, Phalombe | 1,636,879 | 129 | 7.9 | 273 | 16.7 | 402 |
| EU 4 | Machinga only | 481,440 | 26 | 5.4 | 167 | 34.7 | 193 |
| EU 5 | Balaka, Mangochi | 1,231,472 | 40 | 3.2 | 503 | 40.8 | 543 |
| EU 6 | Dedza,  Ntcheu | 1,124,989 | 90 | 8.0 | 93 | 8.3 | 183 |
| EU 7 | Lilongwe | 2,088,989 | 184 | 8.8 | 885 | 42.4 | 1069 |
| EU 8 | Mchinji only | 455,475 | 53 | 11.6 | 102 | 22.4 | 155 |
| EU 9 | Ntchisi Nkhotakota, Dowa only | 1,198,675 | 63 | 5.3 | 95 | 7.9 | 158 |
| EU 10 | Karonga, Rumphi | 461,184 | 83 | 18.0 | 443 | 96.1 | 526 |
| EU 11 | Mzimba,  Nkhata Bay | 1,114,020 | 145 | 13.0 | 162 | 14.5 | 307 |
| Total |  | **11,724,118** | **1318** | **11.2** | **3890** | **33.2** | **5208** |

Note. The population at risk estimates were based on LF programme information as part of the mass drug administration activities in 2011. Not all district level data were available or collected.

**Table B. Summary of clinical cases and prevalence by 100,000 population by districts and sub-district (Traditional Areas) based on active patient searching activities 2014 - 2021**

| District | Traditional Area (TA) name | Population Size | Both | Lymphoedema cases | Hydrocele  cases | LF Clinical Cases | Cases (per 100,000 population) |
| --- | --- | --- | --- | --- | --- | --- | --- |
| Balaka | Balaka Town | 36308 |  | 23 | 13 | 36 | 99 |
| Dedza | Dedza Boma | 30928 |  |  | 1 | 1 | 3 |
| Dowa | Dowa Boma | 7135 |  | 17 | 6 | 23 | 322 |
| Kasungu | Kasungu Boma | 58653 |  | 10 | 7 | 17 | 29 |
| Mangochi | Mangochi Town | 53498 |  | 52 | 28 | 80 | 150 |
| Mchinji | Mchinji Boma | 28011 |  | 28 | 14 | 42 | 150 |
| Mangochi | Monkey Bay Urban | 14955 |  | 58 | 15 | 73 | 488 |
| Nsanje | Mwabvi Game Reserve | 8746 |  | 74 | 5 | 79 | 903 |
| Mwanza | Mwanza Boma | 18039 |  | 27 | 18 | 45 | 249 |
| Mzimba | Mzimba Boma | 26096 | 2 | 69 | 30 | 101 | 387 |
| Salima | Salima Town | 36789 |  | 8 | 13 | 21 | 57 |
| Rumphi | STA Chapinduka | 3754 |  |  | 1 | 1 | 27 |
| Rumphi | STA Chisovya | 9234 |  | 4 | 8 | 12 | 130 |
| Mchinji | STA Gumba | 16953 |  | 31 | 10 | 41 | 242 |
| Mchinji | STA Pitala | 4736 |  | 16 | 2 | 18 | 380 |
| Rumphi | STA Zolokere | 6307 |  | 1 | 2 | 3 | 48 |
| Thyolo | TA Changata | 41828 |  | 39 | 11 | 50 | 120 |
| Chikwawa | TA Chapananga | 88641 | 3 | 229 | 71 | 303 | 342 |
| Ntchisi | TA Chikho | 24711 |  | 11 | 2 | 13 | 53 |
| Zomba | TA Chikowi | 64469 | 2 | 103 | 27 | 132 | 205 |
| Rumphi | TA Chikulamayembe | 70421 | 1 | 54 | 23 | 78 | 111 |
| Ntchisi | TA Chilooko | 89619 | 1 | 24 | 14 | 39 | 44 |
| Nsanje | TA Chimombo | 12334 |  | 24 | 13 | 37 | 300 |
| Mangochi | TA Chimwala | 93858 |  | 122 | 31 | 153 | 163 |
| Mzimba | TA Chindi | 161938 | 3 | 124 | 38 | 165 | 102 |
| Phalombe | TA Chiwalo - Phalombe | 43933 | 1 | 57 | 17 | 75 | 171 |
| Dowa | TA Chiwere | 105708 | 1 | 74 | 46 | 121 | 114 |
| Mangochi | TA Chowe | 112155 |  | 9 | 2 | 11 | 10 |
| Mchinji | TA Dambe - Mchinji | 62248 |  | 19 | 13 | 32 | 51 |
| Ntcheu | TA Goodson Ganya | 143536 | 2 | 155 | 44 | 201 | 140 |
| Phalombe | TA Jenala | 88237 | 1 | 90 | 38 | 129 | 146 |
| Lilongwe | TA Kabudula | 158430 | 1 | 201 | 32 | 234 | 148 |
| Nkhatabay | TA Kabunduli | 50533 | 1 | 28 | 15 | 44 | 87 |
| Dedza | TA Kachere | 167652 | 4 | 71 | 62 | 137 | 82 |
| Phalombe | TA Kaduya | 79357 |  | 11 | 1 | 12 | 15 |
| Ntchisi | TA Kalumo | 74850 |  | 57 | 23 | 80 | 107 |
| Mzimba | TA Kampingo Sibande | 68719 |  | 12 | 7 | 19 | 28 |
| Nkhotakota | TA Kanyenda | 116546 |  | 81 | 26 | 107 | 92 |
| Kasungu | TA Kaomba | 51295 |  | 6 | 4 | 10 | 19 |
| Blantyre | TA Kapeni | 103742 | 2 | 53 | 68 | 123 | 119 |
| Mchinji | TA Kapondo | 10626 | 1 | 19 | 4 | 24 | 226 |
| Salima | TA Karonga | 78297 |  | 32 | 8 | 40 | 51 |
| Ntchisi | TA Kasakula | 19680 |  | 16 | 7 | 23 | 117 |
| Chikwawa | TA Kasisi | 23266 | 3 | 225 | 131 | 359 | 1543 |
| Mangochi | TA Katuli | 71100 | 1 | 53 | 6 | 60 | 84 |
| Rumphi | TA Katumbi | 16028 |  | 4 | 2 | 6 | 37 |
| Machinga | TA Kawinga | 92144 | 1 | 89 | 14 | 104 | 113 |
| Mchinji | TA Kazyozyo | 6971 |  | 7 | 1 | 8 | 115 |
| Karonga | TA Kilupula | 78424 | 9 | 166 | 127 | 302 | 385 |
| Karonga | TAKyungu,Karonga Town | 151281 | 1 | 254 | 80 | 335 | 221 |
| Chikwawa | TA Lundu - Chikwawa | 62176 |  | 68 | 32 | 100 | 161 |
| Chikwawa | TA Makhwira | 79933 | 2 | 180 | 61 | 243 | 304 |
| Nsanje | TA Malemia - Nsanje | 22437 | 2 | 110 | 52 | 164 | 731 |
| Ntchisi | TA Malenga | 50687 |  | 11 | 4 | 15 | 30 |
| Chikwawa | TA Maseya | 37793 |  | 48 | 21 | 69 | 183 |
| Mchinji | TA Mavwere | 83986 | 2 | 33 | 19 | 54 | 64 |
| Lilongwe | TA Mazengera | 116352 | 1 | 145 | 57 | 203 | 174 |
| Nsanje | TA Mbenje | 53559 | 2 | 198 | 44 | 244 | 456 |
| Mchinji | TA Mduwa | 66854 |  | 45 | 18 | 63 | 94 |
| Mchinji | TA Mkanda | 75328 |  | 66 | 22 | 88 | 117 |
| Phalombe | TA Mkhumba | 110623 | 2 | 72 | 53 | 127 | 115 |
| Neno | TA Mlauli | 35569 |  | 20 |  | 20 | 56 |
| Nsanje | TA Mlolo | 69110 | 5 | 241 | 63 | 309 | 447 |
| Machinga | TA Mlomba | 62263 |  | 10 |  | 10 | 16 |
| Mzimba | TA M'Mbelwa | 128656 | 1 | 101 | 49 | 151 | 117 |
| Mangochi | TA Mponda | 167313 | 3 | 138 | 47 | 188 | 112 |
| Dowa | TA Msakambewa | 91421 | 2 | 80 | 30 | 112 | 123 |
| Balaka | TA Msamala | 80912 | 1 | 92 | 29 | 122 | 151 |
| Rumphi | TA Mwahenga | 20067 |  | 24 | 7 | 31 | 154 |
| Karonga | TA Mwakaboko | 24889 | 3 | 83 | 51 | 137 | 550 |
| Rumphi | TA Mwalweni | 27976 |  | 40 | 26 | 66 | 236 |
| Rumphi | TA Mwamlowe | 8481 |  | 16 | 4 | 20 | 236 |
| Rumphi | TA Mwankhunikira | 25466 |  | 51 | 24 | 75 | 295 |
| Karonga | TAMwirang'ombe,TA Wasambo | 110434 | 7 | 250 | 61 | 318 | 288 |
| Mzimba | TA Mzikubola | 99994 |  | 90 | 33 | 123 | 123 |
| Mzimba | TA Mzukuzuku | 37528 |  | 48 | 28 | 76 | 203 |
| Mangochi | TA Nankumba | 159654 |  | 168 | 38 | 206 | 129 |
| Phalombe | TA Nazombe | 60745 |  | 39 | 27 | 66 | 109 |
| Nsanje | TA Ndamera | 33679 |  | 43 | 13 | 56 | 166 |
| Chikwawa | TA Ngabu - Chikwawa | 164753 | 3 | 251 | 58 | 312 | 189 |
| Nsanje | TA Ngabu - Nsanje | 13381 |  | 51 | 25 | 76 | 568 |
| Mulanje | TA Nkanda | 128070 | 1 | 78 | 38 | 117 | 91 |
| Phalombe | TA Nkhulambe | 40313 |  | 31 | 19 | 50 | 124 |
| Machinga | TA Nkoola | 54169 |  | 27 | 7 | 34 | 63 |
| Ntchisi | TA Nthondo | 32219 |  | 12 | 12 | 24 | 74 |
| Mchinji | TA Simphasi | 50571 |  | 58 | 20 | 78 | 154 |
| Machinga | TA Sitola | 25138 | 4 | 146 | 35 | 185 | 736 |
| Nsanje | TA Tengani | 41100 |  | 125 | 50 | 175 | 426 |
| Mchinji | TA Zulu | 69069 |  | 48 | 34 | 82 | 119 |
| Thyolo | Thyolo Boma | 7843 | 2 | 59 | 47 | 108 | 1377 |
| Total |  | **5613230** | **84** | **6333** | **2439** | **8856** | **157.8** |

Note. Include pilot study results from 2014.

**Table C. Summary of lymphoedema and hydrocoele cases by age group and gender based on active patient searching activities 2014– 2021.**

| Age Groups | Lymphoedema | | | Hydrocoele | Both | Overall  Total |
| --- | --- | --- | --- | --- | --- | --- |
|  | **No. of cases (column percentage %)** | | | | | |
|  | **Male** | **Female** | **Total** | **Male** | **Male** | **All** |
| <20 | 53 | 69 | 122 (5.0%) | 806 (12.7%) | 2 (2.4%) | 930 (10.5%) |
| 20 - 29 | 67 | 150 | 217 (1.1%) | 265 (4.2%) | 3 (3.6%) | 485 (5.5%) |
| 30 - 39 | 130 | 236 | 366 (15.0%) | 652 (10.3%) | 9 (10.7%) | 1027 (11.6%) |
| 40 - 49 | 177 | 285 | 462 (18.9%) | 1030 (16.3%) | 16 (19.0%) | 1508 (17.0%) |
| 50 - 59 | 137 | 287 | 424 (17.4%) | 1004 (15.9%) | 10 (11.9%) | 1438 (16.2%) |
| >=60 | 290 | 558 | 848 (34/8%) | 2576 (40.7%) | 44 (52.4%) | 3468 (39.2%) |
| Total | **854** | **1585** | **2439** | **6333** | **84** | **8856** |

**Table D. Summary of lymphoedema cases and acute attacks by stage of condition and gender** **based on active patient searching activities 2014 – 2021.**

| Severity of lymphoedema | Lymphoedema | | | Both | Overall  Total |
| --- | --- | --- | --- | --- | --- |
|  | **No. of cases (average no. of acute attacks)** | | | | |
|  | **Male** | **Female** | **Total** | **Male** | **All** |
| Mild | 357 (1.3) | 729 (1.5) | 1107 (1.4) | 21 (1.5) | 1575 (1.4) |
| Moderate | 207 (2.2) | 376 (2.0) | 601 (2.1) | 18 (1.9) | 1238 (2.1) |
| Severe | 100 (2.7) | 142 (2.5) | 249 (2.6) | 7 (1.6) | 632 (2.5) |
| Total | **664 (1.8)** | **1247 (1.7)** | **1911 (1.8)** | **46 (1.7)** | **1857 (1.8)** |
